# “*It’s Like Stealing What Should be Theirs.”* An Exploration of the Experiences and Perspectives of Parents and Educational Practitioners on Hebrew–English Bilingualism for Jewish Autistic Children

**DOI:** 10.1007/s10803-021-05314-z

**Published:** 2021-10-16

**Authors:** David Ariel Sher, Jenny L. Gibson, Wendy V. Browne

**Affiliations:** 1 Faculty of Education, University of Cambridge, 184 Hills Road, Cambridge, CB2 8PQ UK; 2 Gonville and Caius College, Trinity Street, Cambridge, CB2 1TA UK; 3 Present Address: Department of Psychiatry, Warneford Hospital, University of Oxford, Headington, Oxford, OX3 7JX UK

**Keywords:** Autism, Bilingualism, Monolingualism, Hebrew, English, Jewish

## Abstract

Parents of autistic children are routinely advised to raise them monolingually, despite potential negative effects of monolingualism and no evidence of harm from bilingualism. There is, however, limited research on this topic. This study explored experiences and perspectives of educational practitioners (‘practitioners’) and parents of Hebrew–English bilingual autistic children on bilingualism and monolingualism. Using interpretative phenomenological analysis, we explored the experiences of 22 parents and 31 practitioners using both oral and written interviews. The analysis revealed that religious continuity is a crucial factor in bilingual decision-making. Unexpectedly, both practitioners and parents felt that having to adopt a monolingual approach was unjust, in line with conceptions of forced monolingualism. This article recommends awareness training on community languages and research in other communities.

## Introduction

Autistic children face many inequalities and prejudices in their school years. These include stigma (Kaushik, Kostaki, & Kyriakopoulos, 2016), heightened difficulty in accessing health services and their caregivers facing financial burden (Vohra et al., 2014). Autistic children are up to three times more likely to experience school exclusion than children without special educational needs and disabilities (SEND; House of Commons Education Committee, 2019). Autistic students typically have poorer quality friendships (Kasari et al., 2011; Sher & Gibson, 2021) and experience more bullying from peers (Humphrey & Symes, 2011), greater social anxiety and more loneliness (Bauminger, Shulman, & Agam, 2004; Kuusikko et al., 2008).^1^

### Bilingualism and Monolingual Approaches

Although speaking more than one language is the norm in most parts of the world, parents raising autistic children in countries where the majority language group are primarily monolingual often face an information vacuum when deciding whether their autistic children should gain exposure to a second language or not (Hampton et al., 2017; Kay-Raining Bird, Lamond, & Holden, 2012; Yu, 2013).

Forced monolingualism refers to being prevented from gaining competence in a second language, despite one’s family or culture being bilingual (Clyne, 1991, 2005; Öztürk & Howard, 2018). There are many reports of practitioners (including psychiatrists, language pathologists, teachers and psychologists) advising bilingual parents to avoid exposing their autistic child to a second language (Beauchamp & MacLeod, 2017; Kay-Raining Bird et al., 2012). Such advice raises concerns about hampering autistic children’s communal integration (Beauchamp & MacLeod, 2017; Drysdale, van der Meer, & Kagohara, 2015; Hampton et al., 2017; Jegatheesan, 2011; Kay-Raining Bird et al., 2012; Kremer-Sadlik, 2005; Ohashi et al., 2012; Yu, 2013). Therefore, forced monolingualism constitutes an additional, understudied inequality that autistic children face.

Reasons proffered to explain some practitioners’ monolingual preferences include the belief that a second language causes language confusion and that it impedes acquisition of the majority language. However, these views are not supported by research evidence (Hambly & Fombonne, 2012; Jegatheesan, Fowler, & Miller, 2010a; Jegatheesan, Miller, & Fowler, 2010b; Kay-Raining Bird et al., 2012; Kremer-Sadlik, 2005; Ohashi et al., 2012; Uljarevic et al., 2016; Y’Garcia et al., 2012; Yu, 2013). Paradoxically, research suggests there may be several cognitive and social benefits of bilingualism for autistic children (Beauchamp & MacLeod, 2017; Dai et al., 2018; Genesee, Boivin, & Nicoladis, 1996; Lund, Kohlmeier, & Durán, 2017). These include better performance than monolingual peers in total expressive vocabulary assessments (Petersen, Marinova-Todd, & Mirenda, 2012) and on certain set-shifting tasks (Gonzalez-Barrero & Nadig, 2019). In one study, bilingual autistic children were more likely to vocalise and use gestures in comparison to their monolingual counterparts (Valicenti-McDermott et al., 2013).

There are very few studies on the effects of monolingual approaches among autistic children in ethnic minorities (Jegatheesan, 2011; Yu, 2013). Despite the central role Hebrew occupies in the life of millions of Jewish people (Glinert, 2017), the impact of monolingual approaches among autistic children has not been explored in relation to Hebrew–English bilingualism. Research to bridge this literature gap is crucial because children gaining Hebrew proficiency is pivotal in ensuring the continuity of Jewish faith and tradition (Mintz, 1993; Schiff, 1998). The focus of the current research accords with an important ethical imperative (Tesfaye et al., 2019) to provide a voice for understudied ethnic minorities (Atkin & Chattoo, 2006) and is also of relevance to all diasporic groups endeavouring to prevent community language attrition worldwide, such as Spanish speaking communities in the USA (Valdés, 2006).

Practitioners’ propensity to counsel against bilingualism deleteriously affects heritage language maintenance and limits children’s vocabularies (Fahim & Nedwick, 2014). Whilst some communities do not fully abide by practitioners’ monolingual recommendations for autistic children (Jegatheesan, 2011) many parents do and this can sometimes be on the advice received from family and friends (Beauchamp & MacLeod, 2017; Gibson & Katsos, unpublished; Hampton et al., 2017; Kremer-Sadlik, 2005; Yu, 2013). The impact of this is widely relevant, both globally and in the UK. For example, Wales, with two national languages (Welsh and English) has no specialist autism schools delivering education in Welsh (Howard, Gibson, & Katsos, 2019a). This lack of support for bilingualism means parents are forced to choose between their cultural/heritage preference and their autistic child’s scholastic requirements, an unsatisfactory situation alluded to in Welsh education governmental reviews (Roberts, 2017).

Recommendations that autistic children from bilingual families should be raised as monolingual speakers means children are often thereby deprived of opportunities to accustom themselves linguistically and culturally to their community’s social norms (Beauchamp & MacLeod, 2017) and religious life (Jegatheesan et al., 2010a, b). Forced monolingualism may also impair communication between monolingual children and bilingual parents and family, which undermines the child’s ability to interact and build relationships with immediate and extended family (Beauchamp & MacLeod, 2017; Kremer-Sadlik, 2005; Yu, 2013). Furthermore, such recommendations are not sensitive to the aspirations of bilingual parents, who often desire that their autistic children attain bilingual ability (Howard, Gibson, & Katsos, 2020a; Howard, Katsos, & Gibson, 2020b; Kay-Raining Bird et al., 2012; Yu, 2013). The present research answers calls for further research on parental and practitioner perspectives on bilingualism among autistic children in specialist schools and in more diverse linguistic contexts (Howard, Katsos, & Gibson, 2019b, 2020a, b; Howard, Katsos, & Gibson, 2020c). Outcomes of children in specialist schools, in terms of their quality of life and inclusion, differ markedly from those in mainstream schools. For example, autistic children may face less bullying and experience more support and acceptance in specialist schools (Cook, Odgen, & Winstone, 2016; Moore, 2007), yet no study has explored the experiences of bilingual autistic children in specialist schools.

### Hebrew–English Bilingualism in Jewish Schools

Happé and Frith (2020) note that the way in which cultures and different ethnicities affect autistic people has not yet been explored sufficiently. To our knowledge, no prior study has explored Hebrew–English bilingualism and monolingual approaches among Jewish autistic children. The paucity of literature on Hebrew–English bilingualism among autistic children is compounded by the fact that there is no literature specifically exploring Hebrew–English bilingualism among children with SEND. Several Jewish schools specialise in education for autistic children (Anonymous Schools, 2020). Many Jewish schools in the USA and virtually all Jewish schools in the UK provide instruction in Hebrew alongside English, albeit to varying extents (Miller, 2011; Mintz, 1993). The ramifications of Hebrew–English bilingual ability extends to Jewish lifecycle events. For example, reading from the Torah in Hebrew at *bar mitzvah* is important for affiliated English-speaking Jewish families with autistic children (Hyman, 2009; Muskat & Putterman, 2016).

### Importance of Parents’ and Educational Practitioners’ Perspectives

Parents’ language choices, particularly choices surrounding bilingual exposure, appear to have great influence on autistic children’s bilingual expressive vocabulary abilities (Hambly & Fombonne, 2014). In some regards, such as accent, parental influence is greater for autistic children than that of their peers (Baron-Cohen & Staunton, 1994). Parental perspectives are important in Hebrew–English bilingual contexts, as parental attitudes are critical in determining the extent of children’s Hebrew–English bilingual exposure (Ackerman, 1993; Feuer, 2016). Educational practitioners’ (‘practitioners’) perspectives are equally critical as they exercise significant influence over children’s bilingual outcomes (Lee & Oxelson, 2006) and their awareness of autism-related issues is decisive in successful inclusion of autistic pupils (Iadarola et al., 2015; Sansosti & Sansosti, 2012). Indeed, greater understanding of autism among educators improves autistic students’ educational experiences (Hinton, Sofronoff, & Sheffield, 2008; Symes & Humphrey, 2011).

### The Present Study

Due to the lack of research evidence in this area and the potential importance of Hebrew–English bilingualism for the wellbeing and cultural identity of autistic children who are part of the Jewish diaspora, we designed a study to explore this issue in greater depth. We ask two main research questions;

1. What are the experiences and perspectives of educational practitioners and parents of autistic children on Hebrew–English bilingualism and monolingual approaches?
2. What factors do educational practitioners and parents take into account when deciding that their autistic children/students should gain Hebrew–English bilingual ability?

## Methods

### Methodological Approach

Congruent with methodological developments in multi-perspectival research designs in both interpretative phenomenological analysis (IPA) frameworks generally and in autism research specifically (e.g. Hebron, Humphrey, & Oldfield, 2015; Larkin, Shaw, & Flowers, 2019; Makin, Hill, & Pellicano, 2017; Reid, Flowers, & Larkin, 2005), the present study sought to approach the research questions through exploration of experiences of parents and practitioners.

IPA is idiographic, as it explores individuals’ experiences. IPA is deemed particularly appropriate when the aim of the researcher is to gain nuanced and unique insights into participants’ experiences (Smith, Flowers, & Larkin, 2009). Semi-structured interviews are often employed in IPA studies. These comprise short, open-ended questions and allow the researcher to temporarily deviate from the interview schedule to pursue an intriguing point. IPA is appropriate for this study for several reasons. It is useful for exploration of phenomena on which little is known (Reid et al., 2005) and this study explores such a topic. Moreover, IPA has been argued to constitute a participatory, collaborative effort, with considerable involvement from both researcher and participant (Reid et al., 2005; Smith et al., 2009) and thus may more faithfully portray the original meaning of participants. Finally, IPA aims to reduce power imbalances often inherent in researcher-participant power dynamics, by emphasising the expertise of participants on their own experiences; this is particularly pertinent in research concerning autism (Howard et al., 2019a; MacLeod et al., 2017).

A research question regarding children’s own experiences had to be postponed due to the effects of the COVID-19 pandemic. The multi-perspectival design was adopted to bolster methodological rigour through between-group triangulation (Elliott, Fischer, & Rennie, 1999; Smith et al., 2009).

Although semi-structured interviews are often considered the primary IPA method of data collection, in the present study we used both oral semi-structured interviews and written responses to elicit participants’ accounts of their views and experiences. This approach was planned from the start, in line with calls to use multiple modes of participation as a means of adopting an inclusive research approach (Crane, Sesterka, & den Houting, 2020; Nicolaidis et al., 2019). Furthermore, the founders of IPA, Smith, Flowers and Larkin (2009), caution against paradigm ‘methodolatry’ and advise that IPA can include a diversity of approaches. When carrying out the study, the use of written responses became unexpectedly important due to the effects of the COVID-19 pandemic, which precluded travel and in person interviews.

### Researcher Reflexivity

We used a reflexive approach in our research. The first author used a reflexive journal to consider choice of data collection methods, possible limitations and researcher positionality. The first author has completed degrees in psychology and education, is a rabbi and has undertaken qualitative research training and IPA training led by an author of the Smith et al. (2009) IPA textbook. The second researcher has undertaken qualitative research training, has qualifications in psychology and speech and language therapy, and has personal experience of neurodevelopmental difficulties. The third researcher has qualitative research training, obtained qualifications in psychology and has personal and professional experience with individuals on the autistic spectrum.

### Ethical Considerations

Ethical clearance was applied for and granted by the Institutional Ethics Committee. Informed consent was given by all participants. Pseudonyms were used for all participants.

### Recruitment

**Fig. 1:**
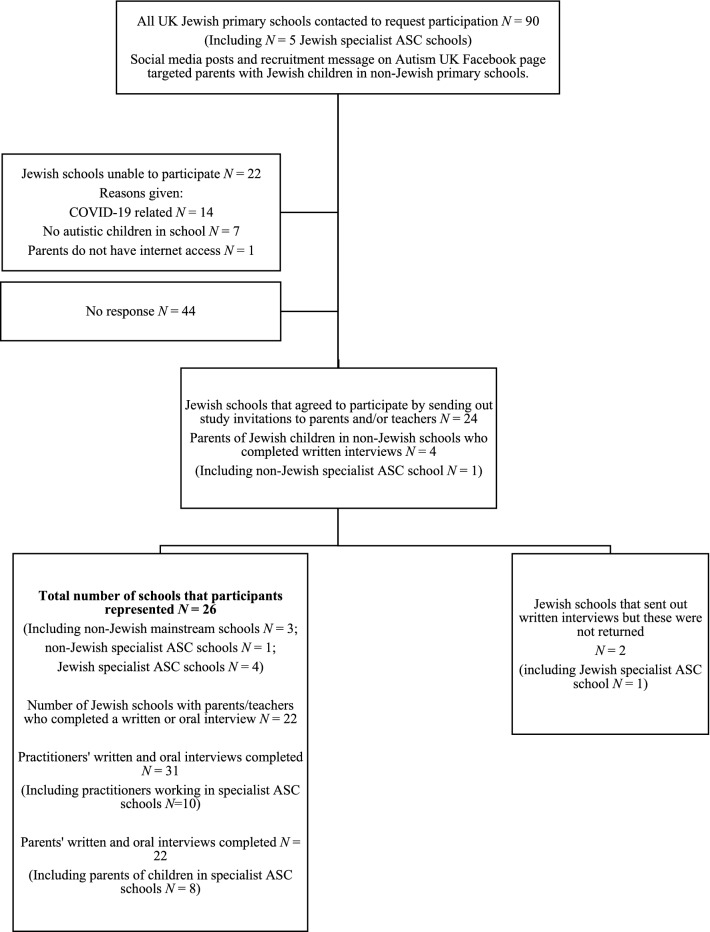
Outline of participant recruitment

The study was based in the UK. All 90 Jewish UK primary schools were contacted via email with information about the study. In total, 24 schools from ten Local Authorities (‘LAs’) across the UK participated by sending invitations to participate to prospective parent and practitioner participants (see Table 1 for school characteristics). Four out of five UK SEND specialist Jewish schools (in three cities) participated, alongside 20 mainstream Jewish schools (see Fig. 1). Participants were recruited via school gatekeepers, who distributed leaflets with information about the study. Schools and participants associated with each of the UK Jewish denominations (strictly-Orthodox, centrist-Orthodox, Masorti, Liberal/Reform) participated.

**Table 1:** Characteristics of schools that distributed study materials to participants

School number	School characteristics *(denomination, SEND/mainstream, single-sex/mixed, state-funded/independent)*	Local Authority
1	Cross communal, mainstream, mixed-gender, state-funded	LA 1
2	Centrist-Orthodox, mainstream, mixed-gender, independent	LA 2
3	Centrist-Orthodox, mainstream, mixed-gender, state-funded	LA 10
4	Centrist-Orthodox, SEND-specialist, mixed-gender, independent	LA 2
5	Centrist-Orthodox, mainstream, mixed-gender, state-funded	LA 2
6	Centrist-Orthodox, SEND-specialist, mixed-gender, independent	LA 3
7	Centrist-Orthodox, mainstream, mixed-gender, state-funded	LA 2
8	Centrist-Orthodox, mainstream, mixed-gender, state-funded	LA 5
9	Cross communal, mainstream, mixed-gender, state-funded	LA 4
10	Centrist-Orthodox, mainstream, mixed-gender, state-funded	LA 6
11	Strictly-Orthodox, mainstream, boys, independent	LA 7
12	Strictly-Orthodox, mainstream, boys, independent	LA 2
13	Strictly-Orthodox, mainstream, girls, independent	LA 7
14	Strictly-Orthodox, mainstream, boys, independent	LA 2
15	Strictly-Orthodox, mainstream, boys, state-funded	LA 7
16	Strictly-Orthodox, mainstream, girls, state-funded	LA 7
17	Strictly-Orthodox, SEND-specialist, mixed-gender, independent	LA 8
18	Strictly-Orthodox, mainstream, boys, independent	LA 8
19	Strictly-Orthodox, mainstream, girls, independent	LA 9
20	Strictly-Orthodox, SEND-specialist, mixed-gender, independent	LA 9
21	Strictly-Orthodox, mainstream, boys, independent	LA 9
22	Strictly-Orthodox, mainstream, boys, independent	LA 9
23	Strictly-Orthodox, mainstream, mixed-gender, state-funded	LA 6
24	Strictly-Orthodox, mainstream, mixed-gender, state-funded	LA 9

LAs and school names are anonymised to protect the anonymity of participating schools

This study sought views from educational practitioners (practitioners) and parents, using the following inclusion criteria:

Practitioners,

1. had direct involvement in the providing education for a child with a formal autism diagnosis, and
2. worked in a school offering Hebrew–English bilingual instruction.

Parents,

1. were guardians of children with a formal autism diagnosis, and
2. had already decided whether their child(ren) would gain Hebrew–English bilingual exposure.

Based on previous research and suggested sample sizes for phenomenological studies (Guest, Bunce, & Johnson, 2013; Howard et al., 2019b, 2020a, b; Morgan et al., 2002), we aimed to recruit 15 practitioners and 15 parents.

### Procedures and Materials

Due to COVID-19 precluding in-person interviewing, participants were offered the option to have an online interview, or to send responses to questions via electronic or paper formats.

### Oral Responses Option

Participants preferring to talk to the researcher were interviewed via Skype or Zoom software. This virtual, face-to-face, direct engagement with parents via video-call accorded with research finding that parents of autistic children value direct contact with research teams (Fletcher-Watson, Larsen, & Salomone, 2019). The interview schedule featured 14 questions focussed on bilingualism, monolingual approaches and decision-making factors, and was administered in a semi-structured way, allowing interview discretion to follow up on certain points. Questions were open-ended and designed to elicit narratives concerning lived experience (Smith et al., 2009; see “Appendix 1”: ‘Interview Schedule’). Interviews were conducted in English.

### Written Responses Option

Participants preferring to give a written response were sent questions online or on paper copies. Questions were similar to interview questions and were separated into three sections; basic information (e.g. date of birth), information on caregivers/practitioners (e.g. level of education) and religious and language background (e.g. questions relating to Hebrew and bilingualism). Virtually all questions were recorded in open-ended text-boxes (online) or open-ended ‘spaces’ (paper; see “Appendix 2”: ‘Written Response Open-Ended Questions’). Due to the fact that for participants completing written responses, we could not probe further or pursue interesting points (as we did orally), we made the decision to include more questions in the written response option. This resulted in more substantial written responses. This assisted when reporting results, as the extra questions facilitated a broadly comparable level of detail to that of oral responses.

### Data Preparation and Analysis

Shortly after the online interviews took place, recordings were transcribed verbatim and false starts, pauses and ‘*guggles*’ (encouraging sounds made by the researcher) were noted, to contextualise the transcription. For both written and oral interview transcripts, each transcript was read three times and annotations were made in the margins. This process was reiterated until the researcher was content that all potential themes had been recorded (see Table 2).

**Table 2:** Outline of stages of IPA analysis employed in this study

Stage	Elucidation of Stage
Stage 1: Reading and re-reading transcripts	Several attentive examinations of one transcript. Enables ‘immersing’ oneself in the data
Stage 2: Writing initial comments and notes in left-hand margin of transcript	Exploratory notation on key issues for participant in relation to the phenomenon/phenomena. Annotation includes conceptual, linguistic and descriptive comments in an analytical style. Researcher strives to understand what participant’s language means to researcher and participant
Stage 3: Noting themes in right-hand margin of transcript	Reduction of voluminous notations to succinctly worded themes. Researcher is nonetheless cautious to preserve complexity of participant’s account and avoid oversimplification
Stage 4: Searching for links across themes	Scrutinise themes. Form clusters of themes through ‘subsuming’ themes under headings that then lead to creation of super-ordinate themes. Appraise which themes are more prominent and/or frequent or pivotal
Stage 5: Repetition of procedure across all transcripts	The process explicated in stages 1–4 are repeated for each transcript. Equal attention must be expended for analysis of each transcript. Each transcript should be explored rigorously and as a distinct case from the former ones
Stage 6: Searching for patterns and super-ordinate themes across transcripts	Patterns across transcripts are searched for. Researcher determines which themes appear most potent. Identify recurring super-ordinate themes. Super-ordinate themes that are not present in over half of the sample may be discarded. A final master list of super-ordinate themes is presented (see Table 6)

Once all themes had been identified, superfluous duplicate themes were removed to create a final master list of superordinate themes. At this point, two independent researchers reviewed the documentation relating to the themes to assess whether the themes corresponded well and had been coherently abstracted from the data.

### Member-Checking

This study employed member-checking, so participants received copies of their transcripts and were offered the opportunity to modify or add to the information they had provided.

## Results

### Participants

Fifty-three participants took part in the study. Ten participants were interviewed orally (5 parents, 5 practitioners), while 43 participants responded in written form (17 parents and 26 practitioners) (see Tables 3, 4 for participant characteristics and Table 5 for group level summary data). The number of participants was higher than expected, perhaps as a consequence of the multi-modal response options and the perceived importance of the issue in the community.

**Table 3:** Parent participant characteristics

Name, Gender, Region	Additional diagnoses	Relationship to child	Levels of education completed by participant	Jewish denomination and level of observance of Jewish religious law	Languages spoken by participant, Participants’ ability to read, write, speak and understand Hebrew	Autistic child’s age and gender	School attended by autistic child	Child’s ability in Hebrew	Method of interview
1. Anthony, Male, North-East	N/A	Father	Primary and secondary school	Strictly-Orthodox Strictly-Observant	English. Yiddish. Can speak, read, write and understand Hebrew	1 Male, 8 years 1 Female, 15 years	Jewish, strictly-Orthodox, North-East, specialist ASC school	Children are capable in reading, writing and translating Hebrew	Oral
2. Denise, Female, North-West	Global Developmental Delay (GDD)	Mother	Primary and secondary school	Strictly-Orthodox Strictly-Observant	English. Can read, write and understand Hebrew	1 Male, 8 years	Jewish, strictly-Orthodox, North-West, specialist ASC school	Child is non-verbal but mother believes child can recognise Hebrew	Oral
3. Jason, Male, North-West	N/A	Father	Primary and secondary school, Bachelor’s degree, teaching diploma	Strictly-Orthodox Strictly-Observant	English. Some French. Can speak, read, write and understand Hebrew	1 Female, 8 years 1 Female, 15 years	Jewish, strictly-Orthodox, North-West, mainstream school	Some ability in reading, writing and translating Hebrew	Oral
4. Lucille, Female, South-West	N/A	Mother	Primary and secondary school, studying at university	Centrist-Orthodox Strictly-Observant	English. Can speak, read, write and understand Hebrew	1 Male, 7 years	Jewish, centrist-Orthodox, South-East, mainstream school	Some ability in reading and writing Hebrew. Superior (above neurotypical average) in speaking Hebrew	Oral
5. Joshua, Male, North-West	N/A	Father	Primary and secondary school	Strictly-Orthodox Strictly-Observant	English, Yiddish. Can speak, read, write and understand Hebrew	1 Male, 5 years 1 Female, Age unspecified	Jewish, strictly-Orthodox, North-West, specialist ASC school	Moderate ability in reading and writing Hebrew	Oral
6. Adelaide, Female, North-West	ADHD, Sensory processing disorder	Mother	Primary and secondary school, Bachelor’s degree	Centrist/ Modern Orthodox Observant	English. Can speak, read and write Hebrew well. Struggles to understand Hebrew	1 Male, 11 years	Non-Jewish, North-West mainstream school	Mediocre ability in reading, writing and speaking Hebrew	Written
7. Susannah, Female, South-West	N/A	Mother	Primary and secondary school	Strictly-Orthodox Strictly-Observant	English. Can read, write and understand Hebrew. Struggles to speak Hebrew	1 Male, 7 years	Non-Jewish, South-West specialist ASC school	No ability in Hebrew	Written
8. Laura, Female, South-West	ADHD, Dyslexia Dyspraxia	Mother	Primary and secondary school, Bachelor’s degree, Law qualification	Traditional Somewhat observant	English. Struggles to read and write Hebrew. Cannot speak or understand Hebrew	1 Female, 16 years	Non-Jewish, South-West, mainstream school	No ability in Hebrew	Written
9. Michelle, Female, South-West	N/A	Mother	Primary and secondary school, business diploma	Traditional Somewhat observant	English. Reads Hebrew well. Struggles to write, speak and understand Hebrew	1 Male, 14 years	Non-Jewish, South-West, mainstream school	Can read Hebrew well. Cannot speak, write or understand Hebrew	Written
10. Alexander, Male, South-West	N/A	Father	Primary and secondary school	Strictly-Orthodox Strictly-Observant	English. Can read and write Hebrew very well. Speaks and understands Hebrew well	1 Male, 7 years	Jewish, centrist-Orthodox, South-West, specialist ASC school	No ability in Hebrew	Written
11. Sarit, Female, South-West	ADHD, Dyslexia	Mother	Primary and secondary school, Teaching diploma	Centrist-Orthodox/Traditional Strictly-Observant	English. Can read, write, speak and understand Hebrew very well	1 Male, 10 years	Jewish, centrist-Orthodox, South-West, specialist ASC school	Cannot read or write Hebrew. Struggles to speak or understand Hebrew	Written
12. Zelda, Female, North-West	ADHD	Mother	Primary and secondary school	Strictly-Orthodox Strictly-Observant	English. Can read and write Hebrew very well. Understands Hebrew well. Cannot speak Hebrew	1 Male, 7 years	Jewish, strictly-Orthodox, North-West, specialist ASC school	Struggles to read and write Hebrew. Cannot speak or understand Hebrew	Written
13. Yvonne, Female, North-West	N/A	Mother	Primary and secondary school, Bachelor’s, Master’s degrees and Doctorate	Strictly-Orthodox Strictly-Observant	English. Can read and write Hebrew very well. Understands and speaks Hebrew well	1 Male, 10 years	Jewish, strictly-Orthodox, North-West, mainstream school	Can read and write Hebrew well. Struggles to understand Hebrew. Cannot speak Hebrew	Written
14. Jessica, Female, North-West	22q11.2 Deletion Syndrome	Mother	Primary and secondary school, Bachelor’s degree	Centrist-Orthodox Strictly-Observant	English. French. Reads Hebrew well Writes Hebrew very well. Struggles to speak or understand Hebrew	1 Female, 11 years	Jewish, centrist-Orthodox, North-West, mainstream school	Can read and write Hebrew well. Cannot speak or understand Hebrew	Written
15. Louise, Female, South-West	ADHD	Mother	Primary and secondary school, completing Bachelor’s degree	Centrist-Orthodox Strictly-Observant	English. Can read and write Hebrew very well. Can speak and understand Hebrew well	1 Male, 7 years	Jewish, centrist-Orthodox, South-West, mainstream school	Struggles to read and write Hebrew Can speak and understand Hebrew very well	Written
16. Rebecca, Female, South-West	ADHD, Pathological Demand Avoidance (PDA)	Mother	Primary and secondary school Bachelor’s and Master’s degrees and Doctorate	Traditional Observant	English. Struggles to read Hebrew. Cannot write, speak or understand Hebrew	1 Male, 7 years	Jewish, centrist-Orthodox, South-West, mainstream school	No ability in Hebrew	Written
17. Trudy, Female, South-West	Dyspraxia	Mother	Primary and secondary school Bachelor’s degree	Traditional Observant	English. Can read and write Hebrew very well. Struggles to speak or understand Hebrew	1 Male, 11 years	Jewish, centrist-Orthodox, South-West, mainstream school	Struggles to read and write Hebrew Cannot speak or understand Hebrew	Written
18. Vera, Female, North-East	N/A	Mother	Primary and secondary school, Bachelor’s degree, Counselling diploma	Strictly-Orthodox Strictly-Observant	English. Afrikaans Reads, speaks and understands Hebrew well. Writes Hebrew very well	1 Male, 17 years	Jewish, strictly-Orthodox, North-East, mainstream school	Can read, write, speak and understand Hebrew well	Written
19. Chantel, Female, North-West	N/A	Mother	Primary and secondary school	Strictly-Orthodox Strictly-Observant	English. Reads and writes Hebrew very well. Understands Modern Hebrew well. Struggles to understand Biblical Hebrew	1 Male, 10 years	Jewish, centrist-Orthodox, North-West, mainstream school	Can read Hebrew very well. Writes Hebrew well. Struggles to understand Hebrew. Cannot speak Hebrew	Written
20. Michaela, Female, North-West	Dyslexia	Mother	Primary and secondary school	Strictly-Orthodox Strictly-Observant	English. Reads Hebrew very well. Writes and understands Hebrew well. Struggles to speak Hebrew	1 Female, 16 years	Jewish, strictly-Orthodox, North-West, mainstream school	Can read Hebrew very well. Struggles to write, speak and understand Hebrew	Written
21. Siobhan, Female, North-West	N/A	Mother	Primary and secondary school	Strictly-Orthodox Strictly-Observant	English. Reads and writes Hebrew very well. Struggles to speak and understand Hebrew	1 Female, 13 years	Jewish, strictly-Orthodox, North-West, mainstream school	Struggles to read and understand Hebrew. Writes Hebrew well, Cannot speak Hebrew	Written
22. Debbie, Female, North-West	N/A	Mother	Primary and secondary school, Fitness diploma	Strictly-Orthodox Strictly-Observant	English. Reads and writes Hebrew very well. Struggles to speak Hebrew. Understands Hebrew to an extent	1 Male, 10 years	Jewish, strictly-Orthodox, North-West, specialist ASC school	Child is non-verbal. Has no ability in Hebrew	Written

**Table 4:** Educational practitioner participant characteristics

Name and position/Gender/Religious denomination/School region, denomination, and type/Method of interview	How many students have an autism diagnosis? Age of autistic student(s)? Gender of autistic student(s)? Additional diagnoses	All levels of education completed by teacher/Countries resided in and years of residence?	All levels of Jewish education completed? Level of religious observance?	Languages autistic students can speak/write or understand? Primary language spoken in classroom?	Number of autistic children who read, write, speak, understand Hebrew? If any, what is their level of proficiency?	How often do autistic students hear Hebrew spoken or read in your classroom? Is this in a ritual context (e.g. prayers) or an everyday spoken language context (e.g. Modern Hebrew)?	How often do your autistic students read Hebrew prayers in your classroom?	How well do you read, write, speak or understand Hebrew?
1. Anne, Teacher/Female, centrist-Orthodox/South-West, Jewish, centrist-Orthodox, mainstream school Written	Students with autism diagnosis: 1/ Age: 4 years/ Male/N/A	Primary and secondary school, Bachelor’s degree, PGCE/ UK “All life”	Jewish primary school/ Observant (Sabbath, Kosher)	Hebrew/Primary language: English	Read Hebrew: Cannot: 0/Write Hebrew: Cannot: 0/Speak Hebrew: Very well: 1 Understand Hebrew: Very well: 1/Unanswered	Approximately once a day/ Mainly prayers (ritual) but there is an *Ivrit* [Modern Hebrew] lesson once a week	Once a day/	Read: well Write: struggle Speak: struggle Understand: struggle/
2. Brittany, Teacher and director of SEND centre/Female, strictly-Orthodox*/*North-West, Jewish, strictly-Orthodox specialist ASC school Written	Students with autism diagnosis: 25 students/ Ages: 6–19 years/ 16 Males, 9 females/ 10 With one of the following: ADHD, Developmental Language Disorder, dyslexia, dyspraxia	Primary and secondary school, Bachelor’s and Master’s degrees/ Israel 3 UK 35	Jewish primary and secondary school/ Seminary/ Strictly observant (all fasts, *Sefira*, Sabbath, Kosher, Family Purity)	English: all students (25)/ French: 2 students/ Yiddish: 7 students/ Primary language: English	Read Hebrew: Very well: 18 Struggle: 5 Cannot read: 2/ Write Hebrew: Very well: 8 Well: 10 Struggle: 5 Cannot: 2/ Speak Hebrew: Very well: 4 Well: 4 Struggle: 2 Cannot: 15/ Understand Hebrew: Very well: 4 Well: 4 Struggle: 15 Cannot 2/	Once a week/ We only use Hebrew words when they come up in a conversation	Never with myself but every day with a religious studies teacher/	Read: very well Write: very well Speak: well Understand: well/
3. Beth, Teaching assistant; key worker for autistic child/Female, strictly-Orthodox/North-West, Jewish, strictly-Orthodox, specialist ASC school Written	Students with autism diagnosis: 2 students/ Ages: 4 years, 8 years/ Both male/ Birthplace(s): UK/ N/A	Primary school/ UK 19	Jewish primary and secondary school, seminary/ Strictly observant (all fasts, *Sefira*, Sabbath, Kosher, Family Purity)	English: 2 students/ Primary language: English	Read Hebrew: Well: 1 Cannot: 1/ Write: Struggle: 1 Cannot: 1/ Speak: Cannot: 2/ Understand: Cannot: 2/	Approximately once a day/ Ritual context	Once a day/	Read: very well Write: very well Speak: very well Understand: very well/
4. Cheryl, Teaching assistant; key worker for autistic child/Female, *Hasidic*/North-West, Jewish, strictly-Orthodox, specialist ASC school Written	Students with autism diagnosis: 2/ Ages: 3 years, 4 years/ Both male/ N/A	Primary and secondary school, childcare diploma/ UK 19	Jewish primary and secondary school, seminary/ Strictly observant (all fasts, *Sefira*, Sabbath, Kosher, Family Purity)	English: 2 students/ Yiddish: 1 student/ Primary language in classroom: English	Read Hebrew: Cannot: 2/ Write: Cannot: 2/ Speak: Cannot: 2/ Understand: Cannot: 2/	Approximately once a day/ Ritual context (“praying”)	Once a day/	Read: struggle Write: cannot Speak: cannot Understand: struggle/
5. Estelle, Teacher/Female, *Hasidic*/North-West, Jewish, strictly-Orthodox, Mainstream school Written	Student(s) with autism diagnosis: 1 student/ Age: 8 years 6 months/ Male/ N/A	Primary and secondary school, Autism and childcare diploma/ UK 52	Jewish primary and secondary school, seminary/ Strictly observant (all fasts, *Sefira*, Sabbath, Kosher, Family Purity)	English: 1 student/ Hebrew: 1 student/ Primary language in classroom: English	Read Hebrew: Struggles: 1 Cannot: 1/ Write: Struggles: 1 Cannot: 1/ Speak: Cannot: 2/ Understand: Cannot: 2/	Approximately once a day/ Ritual context	Once a day/	Read: very well Write: very well Speak: struggle Understand: well/
6. Georgina, Teaching assistant; key worker for autistic child/Female, strictly-Orthodox/ North-West, Jewish, strictly-Orthodox, specialist ASC school Written	Student(s) with autism diagnosis: 1 student/ Age: 10 years/ Male/ Birthplace: UK/ GDD = 1 MEF2C deficiency = 1	Primary and secondary school, AS Level, BTEC, Level 3/ UK 21	Jewish primary and secondary school, seminary/ Strictly observant (all fasts, *Sefira*, Sabbath, Kosher, Family Purity)	English: 1 student/ Signalong: 1 student/ Primary language in classroom: English	Read: Cannot: 1/ Write: Cannot: 1/ Speak: Cannot: 1/ Understand: Cannot: 1/	Approximately once a day/ Ritual context (“only during prayers”)	Once a day/	Read: very well Write: very well Speak: struggle Understand: struggle/
7. Jesse, Headteacher and Rabbi/Male, centrist-Orthodox/South-West, Jewish, centrist-Orthodox, mainstream school Written	Student(s) with autism diagnosis: 4 students/ Ages: 5 years, 8 years, 8 years, 10 years/ All male/ Sensory processing disorder = 4 ADHD = 4	Primary and secondary school, Bachelor’s and Master’s degree/ UK 39 Israel 3	Jewish primary and secondary school, *Yeshiva* (religious college), *Kollel, Semicha* (rabbinic ordination)/ Strictly observant (all fasts, *Sefira*, Sabbath, Kosher, Family Purity)	English: 4 students/ Primary language in classroom: English	Read: Well: 2 Struggle: 2/ Write: Well: 2 Struggle: 2/ Speak: Struggle: 3 Cannot: 1/ Understand: Struggle: 3 Cannot: 1/	Approximately once a day/ Ritual context	Once a day/	Read: very well Write: very well Speak: well Understand: well/
8. Samantha, Headteacher/Female, *Hasidic*/South-West, Jewish, strictly-Orthodox specialist ASC school Written	Student(s) with autism diagnosis: 15 students/ Ages: 4–19 years old/ 13 males, 2 females/ Fragile X syndrome = 1 Down’s Syndrome = 1	Primary and secondary school, Bachelor’s degree, Postgraduate certificate in speech and language/ UK 64	Jewish primary and secondary school, seminary/ Strictly observant (all fasts, *Sefira*, Sabbath, Kosher, Family Purity)	English: 15 students/ Primary language in classroom: English	Read: Struggle: 5 Cannot 10/ Write: Cannot: 15/ Speak: Cannot: 15/ Understand: Cannot: 15/	Several times a day/ Ritual context	Once a day/	Read: very well Write: struggle Speak: struggle Understand: struggle/
9. Geraldine, SENDCo/Female, strictly-Orthodox/North-West, Jewish, strictly-Orthodox, mainstream school Written	Student(s) with autism diagnosis: 4 students/ Ages: 16 years, 16 years, 15 years, 12 years 4 months/ Dyspraxia = 1	Primary and secondary school/ USA 17 UK 22 Israel 1	Jewish primary and secondary school, seminary/ Strictly observant (all fasts, *Sefira*, Sabbath, Kosher, Family Purity)	English: 4 students/ Hebrew: 4 students/ Yiddish: 1 student/ Primary language in classroom: English	Read: Very well: 4/ Write: Very well: 2 Well: 2/ Speak: Very well: 1 Well: 2 Struggle: 1/ Understand: Very well: 1 Well: 2 Struggle: 1/	Several times a day/ Ritual context and in religious studies	Once a day/	Read: very well Write: well Speak: well Understand: very well/
10. Tamlyn, SENDCo/Female, *Hasidic*, strictly-Orthodox*/*North-West, Jewish, strictly-Orthodox, Mainstream school Written	Student(s) with autism diagnosis: 4 students/ Ages: 10 years, 10 years, 12 years, 12 years/ All female/ ADD = 3 DLD = 3	Primary and secondary school, Business management ICC Coaching/ UK 47	Jewish primary and secondary school, seminary/ Strictly observant (all fasts, *Sefira*, Sabbath, Kosher, Family Purity)	English: 4 students/ Hebrew: 4 students/ Primary language in classroom: English	Read: Very well: 2 Well: 2/ Write: Very well: 2 Well: 2/ Speak: Struggle: 4/ Understand: Struggle: 4/	Several times a day/ Scriptural/textual (Ritual)	Once a day/	Read: very well Write: well Speak: very well Understand: very well/
11. Ezra, SENDCo and Rabbi/Male, strictly-Orthodox*/*North-West, Jewish, strictly-Orthodox, mainstream school Written	Student(s) with autism diagnosis: 2 students/ Ages: 10 years, 12 years/ All male/ ADHD = 2	Primary and secondary school/ UK 37 Israel 3	Jewish primary and secondary school, *Yeshiva, Kollel/* Strictly observant (all fasts, *Sefira*, Sabbath, Kosher, Family Purity)	English: 2 students/ Hebrew: 2 students/ Yiddish: 2 students/ Primary language in classroom: English	Read: Struggle: 2/ Write: Struggle: 2/ Speak: Cannot: 2/ Understand: Struggle: 2/	Several times a day/ Ritual	Three times a day/	Read: very well Write: very well Speak: well Understand: very well/
12. Ruth, Teaching assistant/Female, Traditional/North-West, Jewish, Traditional, mainstream school (Traditional, Reform and secular student body) Written	Student(s) with autism diagnosis: 1 student/ Age: 7 years/ Male/ N/A	Unanswered	Jewish primary and secondary school/ Somewhat observant (*Yom Kippur* and *Rosh HaShanah*)	English: 1 student/ Primary language in classroom: English	Read: Struggle: 1 Cannot: 1/ Write: Cannot: 1/ Speak: Cannot: 1/ Understand: Cannot: 1/	Approximately once a day/ Mainly ritual (“prayers”) and sometimes in class learning from Year 2	Never/	Read: well Write: well Speak: struggle Understand: struggle/
13. Rayna, Teaching assistant; key worker for children with SEND/Female, strictly-Orthodox*/*North-West, Jewish, strictly-Orthodox, specialist ASC school (student body: *Hasidic,* strictly-Orthodox, centrist-Orthodox) Written	Student(s) with autism diagnosis: 3 students/ Ages: 3 years, 4 years, 4 years/ Male/ ADHD = 1	Primary and secondary school, Bachelor’s degree, BTECs/ UK 20	Jewish primary and secondary school, seminary/ Observant (Sabbath, Kosher)	English: 3 students/ Hebrew: 1 student/ Yiddish: 2 students/ Primary language in classroom: English	Read: Struggle: 1 Cannot: 2/ Write: Struggle: 1 Cannot: 2/ Speak: Struggle: 3/ Understand: Well: 2 Struggle: 1/	Several times a day/	Never/	Read: well Write: very well Speak: well Understand: well/
14. Sharon, Teaching assistant; key worker for children with SEND/Female, strictly-Orthodox*/*North-West, Jewish, strictly-Orthodox, specialist ASC school Written	Student(s) with autism diagnosis: 3 students/ .Ages: 6 years, 3 years, 4 years/ 2 Males, 1 female/ ADHD = 1 DLD = 2	Primary and secondary school, Childcare diploma/ Mexico 9 UK 13	Jewish primary and secondary school, seminary/ Strictly observant (all fasts, *Sefira*, Sabbath, Kosher, Family Purity)	English: 3 students/ Hebrew: 2 students/ Primary language in classroom: English	Read: Struggle: 1 Cannot: 2/ Write: Struggle: 1 Cannot: 2/ Speak: Cannot: 3/ Understand: Cannot: 3/	Approximately once a day/	Three times a day/	Read: very well Write: very well Speak: very well Understand: well/
15. Barbara, Teacher; key worker for children with SEND/Female, strictly-Orthodox*/*North-West, Jewish, strictly-Orthodox, mainstream school Written	Student(s) with autism diagnosis: 2/ Ages: Uncertain/ Male/ N/A	Primary and secondary school, Teaching diploma Play therapy/ Switzerland 20 UK 22	Jewish primary and secondary school, seminary/ Strictly observant (all fasts, *Sefira*, Sabbath, Kosher, Family Purity)	English: 2 students/ Hebrew: 2 students/ Primary language in classroom: English	Read: Very well: 2/ Write: Well: 2/ Speak: Cannot: 2/ Understand: Well: 2/	Several times a day/ Ritual	Once a day/	Read: very well Write: very well Speak: struggle Understand: well/
16. Lauren, Teacher/Female, *Hasidic/*North-West, Jewish, centrist-Orthodox, mainstream school Written	Student(s) with autism diagnosis: 2/ Ages: 9 and 10 years/ Male/ N/A	Primary and secondary school, Bachelor’s degree, teaching diploma/ UK 44	Jewish secondary school, seminary/ Strictly observant (all fasts, *Sefira,* Sabbath, Kosher, Family Purity)	English: 3 students/ Hebrew: 2 students/ Primary language in classroom: English	Read: Very well: 2/ Write: Very well: 1 Well: 1/ Speak: Well: 1 Cannot: 1/ Understand: Very well: 1 Struggle: 1/	Approximately once a day/ Ritual: daily As an everyday spoken language: once a week	Once a day/	Read: very well Write: very well Speak: very well Understand: well/
17. Victoria, Headteacher/Female, centrist-Orthodox, Traditional/North-West, Jewish, centrist-Orthodox, mainstream school Written	Student(s) with autism diagnosis: 5/ Ages: 3–8 years/ 4 Males, 1 female/ N/A	Primary and secondary school, Bachelor’s degree, NPQH/ UK 55	Jewish primary school/ Observant (Sabbath, Kosher)	English: 5 students/ Modern Hebrew: 5 students/ Primary language in classroom: English	Read: Well: 2 Struggle: 2/ Write: Well: 3 Struggle: 2/ Speak: Well: 3 Cannot: 2/ Understand: Well: 3 Struggle: 2/	Approximately once a day/ Ritual (daily prayers) Modern Hebrew everyday spoken language lessons (once a week)	Once a day/	Read: struggle Write: struggle Speak: struggle Understand: struggle/
18. Stephany, Teacher/Female, centrist-Orthodox, Traditional/North-West, Jewish, centrist-Orthodox, mainstream school Written	Student(s) with autism diagnosis: 1/ Age: 7 years/ Male/ ADHD = 1	Primary and secondary school, Bachelor’s degree, PGCE/ UK 23	Jewish primary school/ Somewhat observant (*Yom Kippur* and *Rosh HaShanah)*	Hebrew: 1 student/ English: 1 student/ Primary language in classroom: English	Read: Very well: 1/ Write: Very well: 1/ Speak: Very well: 1/ Understand: Very well: 1/	Approximately once a day/ Modern Hebrew everyday spoken language	Once a day/	Read: well Write: well Speak: well Understand: well/
19. George, Teacher/ Male, centrist-Orthodox/North-West, Jewish, centrist-Orthodox, mainstream school Written	Student(s) with autism diagnosis: 2/ Age: Uncertain/ 2 male/ N/A	Primary and secondary school, Bachelor’s degree/ UK	Jewish primary and secondary school, *Yeshiva/* Strictly observant (all fasts, *Sefira*, Sabbath, Kosher, Family Purity)	Unanswered Primary language in classroom: English	Read: Struggle: 2/ Write: Well: 2/ Speak: Struggle:2/ Understand: Struggle: 2/	Approximately once a day/ Unanswered	Once a day/	Read: very well Write: very well Speak: very well Understand: very well/
20. Jemimah, SENDCo/Female, strictly-Orthodox/South-West, Jewish, strictly-Orthodox, mainstream school Written	Student(s) with autism diagnosis: 1/ Age: 7 years/ Male/ N/A	Primary and secondary school. Postgraduate diploma on SLD/ UK ‘All life’	Jewish primary and secondary school, seminary*, Kollel/* Strictly observant (all fasts, *Sefira*, Sabbath, Kosher, Family Purity)	English: 1 student/ Primary language in classroom: English	Read: Well: 1/ Write: Well: 1/ Speak: Struggle: 1/ Understand: Struggle: 1/	Several times a day/ Ritual	Once a day/	Read: very well Write: very well Speak: struggle Understand: well/
21. Rose, Therapist/Female, strictly-Orthodox*/*North-West, Jewish, strictly-Orthodox, specialist ASC school Written	Student(s) with autism diagnosis: 10/ Ages: 7 years/ 8 Males, 2 females/ ADHD = 4 DLD = 2 Dyslexia = 3	Primary and secondary schools, Play therapy specialist degree Postgraduate Diploma in Autism/ UK – 51 (Scotland 2 England 49)	Jewish primary and secondary school/ Strictly observant (all fasts, *Sefira*, Sabbath, Kosher, Family Purity)	Yiddish: 8 students/ English: 2 students/ Primary language in classroom: English	Read: Struggle: 9/ Write: Cannot: 10/ Speak: Cannot: 10/ Understand: Struggle: 10/	Several times a day/ Ritual context	Once a day/	Read: very well Write: very well Speak: cannot Understand: well/
22. Sandra, Teacher/Female, strictly-Orthodox/South-West, Jewish, strictly-Orthodox, mainstream school Written	Student(s) with autism diagnosis: 1/ Age: 14/ Female/ Birthplaces: UK/ N/A	Primary and secondary schools, Bachelor’s and Master’s degree/ Israel 5 UK remainder	Jewish primary and secondary school, seminary/ Strictly observant (all fasts, *Sefira*, Sabbath, Kosher, Family Purity)	Yiddish: 1 student/ English: 1 student/ Hebrew: 1 student/ Primary language in classroom: English	Read: Well: 1/ Write: Well: 1/ Speak: Struggle: 1/ Understand: Well: 1/ 1 Student	Several times a day/ Ritual context	Once a day/	Read: very well Write: very well Speak: struggle Understand: very well/
23. Nicola, Teacher/Female, strictly-Orthodox*/*North-West, Jewish, strictly-Orthodox, mainstream school Written	Student(s) with autism diagnosis: 1 student/ Age: 11 years/ Female/ N/A	Primary and secondary school, training and education diploma/ Israel 5 UK 15	Jewish primary and secondary school, seminary/ Strictly observant (all fasts, *Sefira*, Sabbath, Kosher, Family Purity)	English: 1 student/ Hebrew: 1 student/ Primary language in classroom: English	Read: Well: 1/ Write: Well: 1/ Speak: Cannot: 1/ Understand: well: 1/	Several times a day/ Ritual context	Once a day/	Read: very well Write: well Speak: very well Understand: very well/
24. Nora, Educational psychologist and teacher/Female, strictly-Orthodox/North-West, Jewish, strictly-Orthodox, mainstream school Written	Student(s) with autism diagnosis: 2 students/ Age: 10 years/ 2 Female/ N/A	Primary and secondary school, Bachelor’s, Master’s degrees, teaching diploma/ South Africa: 31 UK 23	Jewish primary and secondary school, seminary/ Strictly observant (all fasts, *Sefira*, Sabbath, Kosher, Family Purity)	English: 2 students/ Hebrew: 2 students/ Primary language in classroom: English	Read: Well: 1 Struggle: 1/ Write: Well: 1 Struggle: 1/ Speak: Struggle: 1 Cannot: 1/ Understand: Struggle: 1 Well: 1/	Several times a day/ Ritual context	Once a day/	Read: very well Write: very well Speak: well Understand: very well/
25. Carli, Teaching assistant/Female, *Hasidic/*North-West, Jewish, strictly-Orthodox*,* specialist ASC school Written	Student(s) with autism diagnosis: 2 students/ Age: 4 years/ 1 Male, 1 female/ N/A	Primary school/ UK 25	Jewish primary and secondary school, seminary/ Strictly observant (all fasts, *Sefira*, Sabbath, Kosher, Family Purity)	English: 2 students/ Yiddish: 1 student/ Primary language in classroom: English	Read: Cannot: 2/ Write: Cannot: 2/ Speak: Cannot: 2/ Understand: Cannot: 2/	Several times a day/ Ritual context	Three times a day/	Read: very well Write: very well Speak: well Understand: very well/
26. Laurence, SENDCo/Male, Reform/South-West, Jewish, Non-denominational, mainstream school Written	Student(s) with autism diagnosis: 5 students/ Ages: 1 year 5 months, 2 years 7 months, 3 years 9 months, 4 years 10 months, 5 years 11 months/ 4 Males, 1 female/ ADHD = 1	Primary school, secondary school, Bachelor’s degree, National SENDCo award/ South Africa 35 UK 20	Other: state school/ Somewhat observant: (*Yom Kippur* and *Rosh HaShanah*)	English: 5 students/ *Ivrit* (Modern Hebrew): 5 students/ Ghanaian: 1 student/ Primary language in classroom: English	Read: Well: 1 Struggle: 3 Cannot: 1/ Write: Well: 1 Struggle: 3 Cannot: 1/ Speak: Well: 2 Struggle: 3/ Understand: Well: 2 Struggle: 3/	Several times a day/ Ritual and everyday spoken language context	Three times a day/	Read: struggle Write: very cannot Speak: struggle Understand: struggle/
27. Helena, Teacher/Female, strictly-Orthodox/North-West, Works with autistic children in 3 Jewish schools: centrist-Orthodox, strictly-Orthodox, *Hasidic*, all mainstream Oral	Student(s) with autism diagnosis: 7 students/ 5 Males, 2 females Ages: 8–12 years N/A	Primary school, secondary school, Bachelor’s degree Has lived in UK, USA and Israel	Jewish primary and secondary school, seminary/ Strictly observant (all fasts, *Sefira*, Sabbath, Kosher, Family Purity)	English: 5 students/ Hebrew: 5 students/ Primary language in classroom: English	4 Able to read, understand and write Hebrew, 3 struggle with this	Several times a day/ In Centrist-Orthodox school in ritual and everyday spoken language context In strictly-Orthodox and *Hasidic* schools in ritual context only	Once a day in centrist-Orthodox school, Several times a day in strictly-Orthodox and *Hasidic* schools	Read: very well Write: very well Speak: very well Understand: very well
28. Madeleine, Teacher/Female, centrist-Orthodox/South-West, Jewish, centrist-Orthodox, specialist ASC school Oral	Student(s) with autism diagnosis: 26 students/ 4 Females, 22 males Ages: 6–11 years ADHD = 1 DLD = 1 Dyslexia = 1	Primary school, secondary school, Bachelor’s degree Has lived in UK and Israel	Jewish primary and secondary school,/ Strictly observant (all fasts, *Sefira*, Sabbath, Kosher, Family Purity)	English: 26 students/ Hebrew: 20 students/ Primary language in classroom: English	20 Students have some level of understanding 10 Can read Hebrew	Several times a day/ Ritual and everyday spoken language context	Once a day	Read: very well Write: very well Speak: very well Understand: very well
29. Jeremy, Headteacher/Male, strictly-Orthodox/South-West and North-West, Jewish, strictly-Orthodox, mainstream school Oral	Student(s) with autism diagnosis: 2 students/ 2 Males Ages: 10, 11 years N/A	Headteacher; Ofsted inspector, Primary and secondary school, Bachelor’s degree, Master’s degree, Doctorate, PGCE Has lived in UK and Ireland	Jewish primary and secondary school, *Yeshiva, Kollel* Strictly observant (all fasts, *Sefira*, Sabbath, Kosher, Family Purity)	English: 2 students/ Hebrew: 2 students/ Primary language in classroom: English	2 Can read and write Hebrew to an extent. They cannot understand or speak Hebrew	Several times a day/ Ritual context	Three times daily	Read: very well Write: very well Speak: well Understand: very well
30. Ramona, Teaching assistant/Female, strictly-Orthodox/North-West, Jewish, strictly-Orthodox, specialist ASC school Oral	Student(s) with autism diagnosis: 2 students/ 2 Males Ages: 4 years, 8 years ADHD = 1	Primary and secondary school, completing Bachelor’s degree Has lived in UK all life	Jewish primary and secondary school, seminary Strictly observant (all fasts, *Sefira*, Sabbath, Kosher, Family Purity)	English: 1 students/ Hebrew: 1 students/ Primary language in classroom: English	1 child is non-verbal. The other has basic ability in reading and writing Hebrew	Several times a day/ Ritual context	Once a day	Read: very well Write: very well Speak: struggle Understand: struggle
31. Roxanne, SENDCo/Female, strictly-Orthodox/North-West, Jewish, strictly-Orthodox, mainstream school Oral	Student(s) with autism diagnosis: 4 students 2 Males, 2 females Ages: 10 years, 11 years, 13 years, 15 years Dyslexia = 1	Primary and secondary school, Bachelor’s, Diploma in SEND education Has lived in Israel, Argentina and UK	Jewish primary and secondary school, seminary Strictly observant (all fasts, *Sefira*, Sabbath, Kosher, Family Purity)	English: 4 students Hebrew: 4 students Primary language in classroom: English	2 children have average ability in reading and writing Hebrew. 2 struggle with reading and writing Hebrew	Several times a day/ Ritual context	Three times daily	Read: very well Write: very well Speak: very well Understand: very well

**Table 5:** Group level data

Participant group	Region	Additional diagnoses of children	Highest levels of education	Jewish denominations	Languages autistic children somewhat competent in	Autistic child’s age and gender	School attended by autistic children	Children who read/write/speak or understand Hebrew
Parents’ data (N = 22)	South-West = 9 North-West = 11 North-East = 2	ADHD = 6 Dyslexia = 3 Dyspraxia = 2 GDD = 1 Sensory processing disorder = 1 22q11.2 = 1 PDA = 1	Primary and secondary = 9 Bachelor’s = 6 Doctorate = 2 Other (e.g. diploma, currently at university) = 5	Centrist/Modern Orthodox = 5 Traditional = 4 Strictly Orthodox = 13	Primary language English = 23 Primary Language Hebrew = 1 Ability in Hebrew = 14 Ability in English = 24 Ability in Yiddish = 2	Mean age = 10.42 SD = 3.53 Range of ages: 5–17 years Male = 17 Female = 8	Specialist ASC schools = 8 Mainstream schools = 14 Jewish schools = 18 Non-Jewish schools = 4	Reading = 17 Writing = 16 Speaking = 8 Understanding = 12
Practitioners’ data (N = 31)	South-West = 23 North-West = 8	ADD = 3 ADHD = 17 DLD = 9 Dyslexia = 6 Dyspraxia = 2 GDD = 1 MEF2C deficiency = 1 Sensory Processing Disorder = 4 Fragile X Syndrome = 1 Down’s Syndrome = 1	Primary school = 2 High school = 2 Bachelor’s = 5 Bachelor’s and postgraduate diploma = 8 Master’s = 4 Doctorate = 1 Other (e.g. autism diploma) = 8 Unspecified = 1	Centrist Orthodox = 6 Traditional = 1 *Hasidic* = 6 Strictly-Orthodox = 17 Reform = 1	Primary language in classroom English = 31 schools Ability in: Hebrew = 75 English = 133 French = 2 Ghanaian/Twi = 1 Unspecified = 1 Yiddish = 23 Signalong = 1	Range of ages: 1.5–19 years Male = 109 Female = 33	Specialist ASC schools = 93 Mainstream schools = 55 Jewish schools = 148 All practitioners worked in Jewish schools	Reading = 100 Writing = 76 Speaking = 39 Understanding = 99

Several practitioners preferred to list the range of years of their autistic students’ ages, rather than each student’s age. Therefore, the range of years instead of the mean age and standard deviation is presented for the practitioners’ autistic students’ age. Ability in Hebrew and other languages reflected being able to communicate in these languages. Parents and practitioners were asked to indicate such ability among their autistic children/students, alongside children’s primary and secondary languages. ‘Unspecified’ detail reflects participants choosing not to provide this information

### Superordinate and Subordinate Themes

We abstracted 4 superordinate and 11 subordinate themes, from the data (see Table 6).

**Table 6:** Superordinate and subordinate themes

Superordinate theme	Subordinate theme
1. Not Limiting Autistic Children	• Monolingual Approaches an Injustice
	• Monolingualism Alienates from Community and Families
	• Caution Before Advocating Monolingualism
	• Autistic Children Not Different from Others
2. Centrality of Hebrew	• Hebrew Affords Multifaceted Integration
	• Hebrew Supports Positive and Spiritual Identity
3. Differences in Observance	• Emphasis on Hebrew Reflects Extent of Religiosity
	• Orthodox Default Position to Teach Hebrew
4. Decision-Making Factors on Bilingualism	• Many Decision-Making Factors
	• Importance of English
	• Learner’s Capability in Primary Language

#### Superordinate Theme 1: Not Limiting Autistic Children

This superordinate theme reflected participants’ belief that adopting a monolingual approach for autistic children was in general, a retrogressive step. There was a belief that this approach should only be adopted with caution, that it distances children from their relatives and communities, is discriminatory, and often constitutes an injustice.

#### Subordinate Theme: Monolingual Approaches an Injustice

Some parents recounted personal experiences with practitioners to illustrate how a default to monolingualism is experienced as an injustice. Lucille related; *I met a…doctor about Ron’s autism…he said straightaway…two languages with children like this complicates things…he didn’t even know Ron…it was part of the rhetoric…He was over-generalising…I was shocked…(Lucille, parent)* By using the words ‘rhetoric’ and ‘over-generalising’ it appeared that for Lucille, the doctor was somewhat capricious in immediately advocating a monolingual approach.

Parents and practitioners referred to monolingual approaches constituting a wrongdoing due to monolingualism impoverishing children’s engagement with Judaism and their relatives. Some parents went further in using emotive terminology and referred to monolingual approaches as a crime, in depriving children from learning Hebrew scripture; *…all that learning…it’s their heritage at the end of the day. It’s like stealing what should be theirs. (Jason, parent)* This strong sense of injustice was corroborated by other parents; *Without…Hebrew it’s a major handicap…our secondary schools are preparing boys to go to yeshiva* [religious college] …*that takes a very high level of understanding of Hebrew…if you haven’t got those language skills you have a major, major, disadvantage. (Anthony, parent)* The impediment engendered by monoliteracy/monolingualism is emphasised by Anthony’s repetition of a ‘major, major’ disadvantage.

Practitioners’ views echoed those of parents concerning the injustice of assuming monolingualism is the only option for autistic children; *There’s a lot of ignorance…people…who hear that a person has autism…switch to the old-fashioned model…They think* [erroneously] *‘Ooh, they’re retarded, they can’t…*[learn Hebrew]’*…You really need to give every person a chance to shine… (Helena, practitioner)* Here, Helena conveyed the limitations and prejudice of monolingualism, by associating it with an ‘old-fashioned’ approach and offensive terminology. Helena seemed to indicate the injustice by affirming the need to allow every individual to thrive.

#### Subordinate Theme: Monolingualism Alienates from Community and Families

Parents indicated that if children could not learn Hebrew, they would endure significant alienation from their community and family; *It would definitely be very different* [if he did not learn Hebrew]*…he would stick out like a sore thumb…it would probably be embarrassing for him. (Joshua, parent)* Other parents and practitioners concurred; *He would feel alienated* [if he would not learn Hebrew] *because…he wouldn’t know what’s going on…In…school they light the menorah and sing…*[so] *when we light the menorah at home and sing…Hanukkah songs…it’s familiar, he relates, he seems calmer. (Denise, parent)* [At] *synagogue…it would…be difficult for them…if they’ve had no exposure…it would be foreign. (Ramona, practitioner)* Both Ramona and Denise underscore here how exposure to Hebrew can allow children to feel more comfortable when present at Jewish ceremonies or worship and how their children would be distanced from their community and family if they only had ability in English. Denise highlighted this through relating her son’s calm demeanour when taking part in religious ritual at home and the alienation he would experience with no knowledge of Hebrew. Ramona emphasised the alienation that could result from imposing monolingualism; making synagogue worship a foreign experience for autistic children.

#### Subordinate Theme: Caution Before Advocating Monolingualism

Both parents and practitioners believed that caution should be applied before pursuing a monolingual approach; *Don’t limit your child’s future by saying ‘Oh…he has ASD…I may as well teach him English…but it’s too hard to read Hebrew’…Otherwise, you’re just leaving them with an even bigger disability…you’re extending the disability, not minimising it. (Anthony, parent)* Here, Anthony conveyed that well-intentioned efforts that result in a monolingual approach may prove to be pernicious by exacerbating the experience of ‘*disability*’. Helena, a practitioner, similarly related; *I think they* [practitioners] *need to be very cautious…we cannot clip the wings of people…every person who makes a recommendation should really be very, very careful and….not generalise… (Helena, practitioner).*

#### Subordinate Theme: Autistic Children Not Different from Others

Parents asserted they did not comprehend why a distinction between autistic and non-autistic children should be made regarding bilingualism, postulating this was discriminatory. One parent declared; *Why should a child with ASC be different to any other child* [?] *…with correct understanding…adjustments…all children should be able to succeed. (Adelaide, parent)* Practitioners related that they strongly believed that children should not be treated differently from neurotypical children in relation to bilingualism; *The decision-making has to do with…level of academic ability…it has nothing to do with the fact that he’s autistic…I would teach them like I teach any child…it wouldn’t be different. (Helena, practitioner) There’s no reason why a child with autism can’t manage a second language…we see…very clearly that they can. (Anthony, parent)* Other parents and practitioners bolstered this argument by asserting that they believed autistic children (unless they had dyslexia or dyspraxia) could, in many instances, be even more adept at developing bilingual ability than neurotypical children and should not be treated differently to others. To take one example, Joshua substantiated this by relating that his autistic daughter travelled to Israel and “*didn’t speak…a word of Hebrew*” yet within six months “*she was fluent in Hebrew*.” *(Joshua, parent)* As such, the argument that autistic children were different to other children in having lesser linguistic ability was rejected by participants.

#### Superordinate Theme 2: Centrality of Hebrew

The second superordinate theme indicated the central importance of Hebrew in the lives of participants and the autistic children they care for. In their view, Hebrew facilitated integration on many levels and also helped autistic children to create a spiritual and positive identity.

#### Subordinate Theme: Hebrew Affords Multifaceted Integration

In parents’ experiences, Hebrew provided their children with access to seminal Jewish cultural encounters important for religious and communal integration, such as reading from the Torah on their *bar mitzvah* and worship in the synagogue. From both parents’ and practitioners’ perspectives, Hebrew–English bilingualism should be supported, as it plays a pivotal role in facilitating Jewish continuity. When asked what Hebrew meant to them and their families, 15 parent participants indicated that it was critically important in their lives, due to cultural and religious reasons; *“…central to our daily lives.” (Yvonne, parent) A bonding factor between ourselves, our nation and our G-d. (Vera, parent)* This was mirrored amongst practitioners. Through listing numerous ways in which learning Hebrew assists in inclusion and integration, Jason, a parent, effectively conveyed the pivotal importance of the language for Jewish autistic children; *For education…and spirituality…for integration into the community…socially…if they’re not experiencing what other children are experiencing or not able to access those things…they’re…even further away, on the periphery (Jason, parent).*

Within Jewish communities, frequent interspersal of Hebrew words within English discourse was cited as another critical example of the way in which Hebrew serves as a conduit for communal and familial integration. As Denise related, at home, family members say; “*‘Shabbos* [Hebrew word for Jewish Sabbath] *is coming’…we don’t say ‘Saturday is coming’” (Denise, parent)* Denise indicated that understanding Hebrew spoken at home and in school promotes autistic children’s comprehension of Judaic practise. Lucille averred that knowledge of Hebrew enables her son to converse with his cousins and provided him with better social *‘tools’*, which were most important “*because…social situations are difficult for him.”* Thus, Hebrew–English bilingualism is viewed as potentially supportive for those with social difficulties. Practitioners emphasised the contribution Hebrew makes for religious inclusion. It thus seems that Hebrew was more than a means of communication for participants; it is deeply interwoven into the religious, social and cultural fabric of Jewish culture and facilitates integration in these contexts. When asked to share what the experience of choosing whether their child should learn Hebrew or not was like, typical responses were forceful; “*It was a given…didn’t require any thought!” (Chantel, parent).*

#### Subordinate Theme: Hebrew Supports Positive and Spiritual Identity

To most parent participants, Hebrew was important in their autistic child’s formulation of a positive identity. Anthony described how this was so for his son; *My son…has actually learnt…three languages* [English, Hebrew, Yiddish]…*he’s able to do that…no problem at all…learning a language is very powerful for a child with ASD….very empowering…It’s tapping into their strengths… (Anthony, parent).*

The positive influence of mastering Hebrew was also noted by practitioners. For example, Jeremy, a headteacher, conveyed that he had observed that autistic children *…get a sense of achievement…from learning a second language. (Jeremy, practitioner)* This point was echoed by Jason, a parent; *I think as well…there’s a sense of achievement…it’s a level of being able to communicate with God* [in Hebrew]*…Judaism is who we are at the end of the day. (Jason, parent)* By stating Judaism is *“who we are*” in this context, Jason indicated the pivotal role of Hebrew for Jewish identity. Parents opined that a positive Jewish communal identity was achieved through participation in Hebrew synagogue services. Parents felt passionate about this; *YES, no question! Language is a crucial tool…to access shared understanding…Without…specialised words for a cultural, religious or national experience, one cannot achieve…shared understanding…That affects one’s ability to feel ‘the same as’ or ‘part of’* [the community]. *(Vera, parent)* Many practitioners (*n* = 20) similarly felt that not learning Hebrew would severely undermine the formation of a communal, and religious identity. As one stated; “*…it would be…an impediment for life.” (Nicola, practitioner)* Only five parents thought their children’s experiences would not be different without learning Hebrew.

#### Superordinate Theme 3: Differences in Observance

The third superordinate theme revealed how differences in levels of religious observance impacted upon Hebrew–English bilingual language learning. Overall, more religious participants placed greater emphasis upon their autistic children gaining bilingual ability through learning Hebrew. Correspondingly, Orthodox schools adopted a default position of initially attempting to teach Hebrew to all students, regardless of an autistic diagnosis.

#### Subordinate Theme: Emphasis on Hebrew Reflects Extent of Religiosity

All parents indicated that Hebrew was of great importance to their families. However, some parents indicated that this was not fully understood by less religious practitioners. Jason revealed this was because they *“…don’t understand socially what it is…the…different milestones we have in life…” (Jason, parent)* Indeed, both Jason, a parent, and Ramona, a practitioner, disclosed that this could sometimes cause friction between parents and practitioners. As Anthony stated; *…*[Religious] *parents…only accept it* [a monolingual recommendation] *if it came from…a professional who understands*…*it’s extremely emotional…parents want their child to learn 'Aleph-Beis'* [Hebrew alphabet]. *(Anthony, parent).*

Anthony’s account suggested a clash in perspectives, between less religious professionals, who did not comprehend the vital importance of Hebrew in Jewish spiritual life, and religious parents who found it testing to envisage their child not learning Hebrew. Approximately half of practitioners viewed Hebrew as an important factor in parents’ decision-making; *“It is of utmost importance” (Geraldine, practitioner); “Of extreme importance.” (Nicola, practitioner)* Practitioners in strictly-Orthodox schools related that parents viewed Hebrew as central in decision-making. Conversely, practitioners in centrist-Orthodox or cross-communal schools did not discern Hebrew to be as influential in decision-making for parents in their schools. A headteacher of a centrist-Orthodox school opined; “*Not very important…They* [parents] *choose the school for…cultural connections, for social reasons”. (Victoria, practitioner).*

#### Subordinate Theme: Orthodox Default Position to Teach Hebrew

A striking finding was that in many Orthodox schools, the default position is for autistic children to learn Hebrew; *All…children are learning all…studies of Judaism* [including Hebrew]. *(Roxanne, practitioner) The default position is always to learn* [Hebrew]. *(Anthony, parent).*

Similar accounts of this default position were provided by other practitioners. The matter-of-fact way in which Joshua, Roxanne and Anthony described this default policy seemed to reveal that they did not view this position as unusual or problematic.

##### Superordinate Theme 4: Decision-Making Factors on Bilingualism

The final superordinate theme illustrated how participants took many factors into consideration when making decisions on bilingualism for their autistic children. It showed that participants recognised the critical importance of their autistic children gaining proficiency in the dominant societal language, English. It also indicated that participants would generally refrain from teaching autistic children Hebrew if their children were not capable in their primary language, whatever that might be.

##### Subordinate Theme: Many Decision-Making Factors

As will be seen in the explication of this subordinate theme, several factors were deemed important by participants for decision-making on bilingualism. In practitioners' opinion, cognitive ability and emotional well-being, family background and whether bilingualism would affect children’s mastery of their primary language were the most important factors in language maintenance decisions. However, for more observant schools, learning Hebrew took precedence over other factors; “*Our children begin learning Hebrew before…learning English…it is seen as a religious imperative” (Ezra, practitioner)* When queried on what were the most important factors, most parents referred to religious and communal reasons; *There is nothing…more important to us than our religion. (Chantel, parent) Learning Hebrew…allows him to follow in shul* [synagogue]*, allows him…to have a bar mitzvah. Everything we do as a Jewish household…revolves around being able to read Hebrew. (Adelaide, parent)* Similarly, practitioners report that *Some parents…are very particular about their children receiving a Hebrew education and…consider this to be more important than other…factors. (Rayna, practitioner)* When indicating the importance of different factors influencing decision-making on Hebrew–English bilingualism, Jewish continuity was deemed most important by both practitioners and parents. Only four parents and three practitioners viewed the ability to converse with Israelis as an important factor in bilingual decision making. Most practitioners (*n* = 20) reported they had received support in their decision-making on bilingualism from the school and they were satisfied with this advice. A majority of practitioners indicated that generally the school and practitioners advised that children should not be precluded from learning Hebrew; *All children…have the right to be included in all learning. (Laurence, practitioner)* One headteacher indicated; *We are ambitious…for them to achieve like the other children. Our autistic children usually learn Hebrew…like the rest of the class. (Jesse, practitioner).*

Practitioners (*n* = 17) indicated that in the absence of debilitating cognitive ‘impairment’, they would recommend that parents of autistic children attempt to ensure their children learn Hebrew as a second language; *I would…recommend their child learns a second language…because I have seen how these children picked up the language with relative ease…and how integrated…they are. (Geraldine, practitioner)* Most parents (*n* = 18) and practitioners (*n* = 22) who answered the question of whether, in their opinion, autistic children *“should be taught Hebrew in addition to their primary language”,* answered affirmatively.

##### Subordinate Theme: Importance of English

Interestingly, although participants indicated that some Orthodox families teach their children Hebrew before English, Anthony declared; *You would always prioritise English over Hebrew…once…we’re happy* [with] *the English…they’ll get Hebrew as well. (Anthony, parent)* This policy may be due to a more pragmatic approach, as English would be needed for quotidian, mundane activities. Practitioners concurred, as Roxanne, a SENDCo, averred; *If…Hebrew and English are both a problem…I would say to the parents…you are living in England…we have to focus…on one language being learnt properly. (Roxanne, practitioner).*

##### Subordinate Theme: Learner’s Capability in Primary Language

Parents emphasised religious factors over academic concerns when they related their decision-making on whether their child would gain Hebrew–English bilingual ability. However, practitioners were more concerned with cognitive ability, particularly when participants did not have proficiency in their primary language. In fact, some practitioners indicated dangers in teaching Hebrew when the first language was not fully acquired due to cognitive difficulties; *They tried to teach her with so many methods, multi-sensory…the iPad…nothing…she was so frustrated…she’s sitting in the classroom…wants to be like everyone else but…she couldn’t get the Hebrew. (Roxanne, practitioner)* Roxanne’s account clearly exemplified how the school was cautious on this issue, as learning a second language may sometimes cause distress to autistic children who have not mastered their primary language first.

## Discussion

This is the first study to explore views and experiences of Hebrew–English bilingualism among parents and practitioners supporting autistic children.

The superordinate theme ***‘centrality of Hebrew’*** shows that amongst Jewish parents and practitioners, a child’s autism diagnosis does not diminish the importance of the child gaining Hebrew–English bilingual ability. This finding complements other studies that illustrate the significance of Hebrew in Jewish life and may be related to the belief that Hebrew is essential for Jewish continuity (Glinert, 2017; Mintz, 1993; Schiff, 1998). The superordinate theme, ***‘decision-making factors on bilingualism***’ reveals that continuity of Jewish tradition was deemed an extremely important factor in decision-making on Hebrew–English bilingualism by a greater number of practitioner and parent participants than other factors, such as the ability to converse with Israelis. In accordance with Miller (2011), the third superordinate theme, *‘****differences in observance’***, demonstrates that Hebrew occupies a weightier role in more religious families, where it was more crucial for familial and cultural cohesion.

The superordinate theme ‘***not limiting autistic children’*** reflects parents' reports that their autistic children often acquired a second language with relative ease. This accords with the findings of existing literature observing that, compared to their monolingual counterparts, bilingual autistic children do not incur additional language development delays (Drysdale, et al., 2015; Hambly & Fombonne, 2012; Ohashi et al., 2012; Valicenti-McDermott et al., 2013).

On the other hand, while autism was not the deciding factor, the present study sheds new light on other factors influencing decisions about language use. Firstly, as seen in the superordinate theme ***‘decision-making factors on bilingualism***’**,** a child’s cognitive ability is a more influential decision-making factor on bilingualism for practitioners than parents, perhaps because of concerns linked to academic considerations. Most unexpectedly, given previous research reporting a more ambivalent attitude to bilingualism among professionals, practitioners were overwhelmingly in agreement with parents in their view that a default monolingual approach is an injustice. Practitioners reported actively encouraging the study of Hebrew. Crucially, practitioners revealed that the ‘default’ position in their schools is to provide Hebrew–English bilingual instruction for autistic students. This stands in marked contrast to findings of a widespread proclivity of practitioners to advocate a monolingual approach for autistic students (Beauchamp & MacLeod, 2017; Drysdale et al., 2015; Hampton et al., 2017; Ohashi et al., 2012).

Both parents and practitioners indicated that in their experience, many autistic children (unless they had dyslexia or dyspraxia) had superior ability in gaining Hebrew proficiency compared to their neurotypical counterparts. This finding differs from Howard et al.’s (2020b) research in non-Jewish schools, which revealed that some practitioners doubted the feasibility of bilingualism for autistic pupils and maintained that it may have a negative impact on their development. Nevertheless, it should be noted that some practitioners in the present study similarly did not recommend bilingualism for children struggling to master their primary language. Further research could explore decision-making in the context of autistic children’s verbal ability in English compared to their community language in greater detail.

Further, as indicated in the first superordinate theme ***‘not limiting autistic children’***, there was a heightened awareness amongst practitioners of how monolingualism could alienate children from their communities. Similarly, great understanding (as seen in the second superordinate theme, ***‘centrality of Hebrew’***) of how Hebrew facilitated communal, social, religious and scholastic integration and inclusion was manifest amongst parents and practitioners. The discovery that practitioners are cognisant of the importance of community languages to minority families diverges from existing literature (Jegatheesan, 2011; Kay-Raining Bird et al., 2012; Yu, 2013).

Admittedly, parents indicated that speech therapists and paediatricians beyond the Jewish community advised them in accordance with the commonplace monolingual approach that other studies have noted (Hampton et al., 2017). However, surprisingly, unlike other research (Kremer-Sadlik, 2005; Yu, 2013), due to the support of schools and practitioners who encouraged their bilingual preferences, parents did not heed these monolingual recommendations. Only in cases where it was found that students were struggling to master their primary language, practitioners’ and parents’ decision-making concurred in that English should take precedence, as it is crucial for societal engagement. This judgement is mirrored in other communities (Howard et al., 2020a, b).

Potential explanation of the divergence of this study’s findings from the existing literature is that, unlike previous studies, practitioners shared a similar culture to that of the parents of autistic children. As such, it appears there was greater sympathy and understanding towards bilingualism where languages were related to the shared culture. This suggestion is buttressed by the finding in the superordinate theme ‘***differences in observance’***, that parents found that less religious practitioners or those from different cultures advocated a monolingual approach, as they did not fully understand community values. The implication of this appears to be that it is critical for practitioners to develop understanding of the cultures of the families they engage with, potentially through training or awareness courses. Similar suggestions have been made for practitioners engaging with autistic pupils from other ethnic minorities (Howard et al., 2019b; Jegatheesan, 2011). Indeed, Nag et al. (2019) indicated that when educators in lower- and middle-income countries ignore home culture and community artefacts, this alienates parents, often with pernicious results for children’s literacy (Nag, Snowling, & Asfaha, 2016). The present findings also accord with research concerning multilingualism in schools in England, Scotland and Wales (McPake, Tinsley, & James, 2007). Research conducted by the Training and Development Agency (TDA) in England revealed that newly qualified teachers did not feel they had been sufficiently trained to work with students from diverse linguistic and cultural backgrounds. McPake, Tinsley and James (2007) also called for professional development and teacher training which provides knowledge on benefits of plurilingualism. It also seems prudent for schools to at least attempt to match autistic children with practitioners of similar cultural backgrounds. As both parents and practitioners indicated, when this matching occurs, parents’ values concerning their language choices are often respected, as the practitioner is cognisant of the communal and/or religious importance and aspirations attached to the second language.

Kay-Raining Bird, Lamond and Holden (2012) and Yu (2013) found that teachers often perpetuated a ‘deficit’ view on autism, in that they maintained that autism limited students’ abilities, including their ability to learn a second language. However, as indicated by the first superordinate theme, ***‘not limiting autistic children’***, this study did not find this ‘deficit’ view to be present amongst practitioner participants. All practitioners noted that autistic children often performed better in language-related tasks than their neurotypical counterparts. Indeed, alongside parent participants, practitioner participants discussed the injustice of treating autistic children differently with unexpected passion and forcefully conveyed that a monolingual attitude belied a somewhat discriminatory and outdated approach which viewed autism as always constituting severe deficit. In accordance with Howard et al.’s (2019b) findings, both practitioners and parents noted that when autistic children gained Hebrew–English bilingual ability, this was inordinately empowering and positively enhanced their identity formation (see superordinate theme ***‘centrality of Hebrew’***). Therefore, it seems clear from these findings that the widespread ‘deficit’ view among teachers should be challenged, as autistic children successfully gained bilingual ability and this enhanced positive identity formation.

### Strengths and Limitations of the Present Study

This study featured wide participation in cities across the UK and schools representing all strands of Judaism. By employing such space triangulation, this study avoided research ‘parochialism’ in only reflecting one sub-culture or city. A second level of space triangulation was accomplished through data collection in mainstream and specialist autism schools, different stakeholder groups, denominations, and different geographical regions (Campbell et al., 2020; Patton, 1999). This means the research was not geographically, professionally or denominationally bound with respect to the UK context. A further methodological strength lies in the IPA methodology, as this has been regarded as a highly suitable method for a sympathetic exploration of diverse experiences and perspectives due to its employment of a reflexive, interpretivist ‘double hermeneutic’ (Howard et al., 2019a, 2020a, b; Smith, 2004; Smith et al., 2009). Moreover, IPA aims to level the balance of power between interviewees and researchers by viewing participants as experts on their own experiences (MacLeod et al., 2017). This feature is ethically apposite for research involving autistic children’s parents, who have to contend with practitioners’ advice that often runs counter to their own wishes (Hampton et al., 2017).

The fact that only UK-based parents and practitioners participated in this research can be assessed to be one limitation of this study. Additionally, whilst this study did include participants who did not view themselves as particularly religious, a majority were observant of at least some religious tenets. As such, this study could potentially be partially limited in largely reflecting the experiences of somewhat observant Jewish parents and practitioners. Critically though, it should be noted that findings may still reflect the experiences of most Jews in the UK (and countries with similar Jewish demographic compositions, such as South Africa and Australia), for approximately three quarters of the UK Jewish community are nominally Orthodox (Casale Mashiah & Boyd, 2017). It is acknowledged that the researcher could not ask participants to elaborate on written responses in person and so these may not reflect views as richly as interviews. Nevertheless, the effect of this was mitigated by larger numbers of participants and by the fact that many participants wrote very detailed and full accounts. Furthermore, the written responses were bolstered by substantial semi-structured oral interview data, and the study benefitted from having a multimodal response which mitigated the weaknesses inherent in any one method through between-method triangulation (Campbell et al, 2020).

## Conclusions

This study has revealed the necessity of cultural awareness of Hebrew’s importance as a community language for Jewish communities, including their autistic members, a finding which may be of relevance beyond the Jewish community. Where such awareness exists, parents feel more reassured in the decision-making advice offered by SENDCos, teachers and psychologists. Such mutually-respectful understanding facilitates autistic children successfully gaining bilingual ability.

It is clear from the current study that despite a growing body of research indicating attendant benefits of bilingualism for autistic children (Beauchamp & MacLeod, 2017; Uljarevic et al., 2016), the propensity of practitioners from dissimilar cultural backgrounds to recommend a monolingual approach for autistic children persists. It is therefore essential that practitioners are made aware of the concrete positive outcomes of bilingualism for autistic children that this study observes and which previous research has predicted (e.g. Creese et al., 2006; Howard et al., 2019b; Jegatheesan, 2011). More rudimentarily, practitioners should be mindful of the significant impoverishment of social and communicatory experiences that unnuanced monolingual approaches engender and of the concomitant reduced engagement with family, and threat to basic human rights that other studies have also noted (Beauchamp & MacLeod, 2017; Gréaux, Katsos, & Gibson, 2020; Jegatheesan, 2011; Kremer-Sadlik, 2005).

This study has provided a much-needed focus on the experiences of autistic Hebrew–English Jewish bilingual children. This topic has been explored in Chinese (Yu, 2013) and Muslim communities in the USA (Jegatheesan, 2011) and in Welsh-medium schools (Howard et al., 2019b). Nevertheless, more research is needed to reflect the diverse linguistic repertoire of autistic children in many communities. In particular, to guide future practice, further research on children’s perspectives on this topic would be worthwhile, especially research exploring any differences in parents’ and children’s viewpoints. Overall, the present study revealed a community-wide attitude wherein monolingual approaches are not supported or advocated. This observation may serve as a noteworthy contribution to our understanding of this under-researched topic, as it reveals how enhanced cultural understanding of the cruciality of bilingualism to certain communities ensures that parents’ values and children’s integration are both championed and respected.

## Interview Schedule

Interviews accord with Interpretative Phenomenological Analysis (IPA), as elucidated in: Smith, J., Flowers, P., & Larkin, M. (2009). *Interpretative phenomenological analysis: Theory, method and research*. London: SAGE.

1. Can you describe what Hebrew means to you and your family?
2. Can you share what the experience of choosing whether your child should learn Hebrew or not was like?
3. What would you say were the most important factors in making that decision?
4. If you had to put your finger on the most important factor influencing your decision, what would you say that was?
5. Did you experience support in your decision-making from the school, or from professionals? (If so, what form did this take?)
6. Would you say that learning Hebrew would enhance your child’s connection to the wider Jewish community? (If so, in what way?)
7. Would you say that learning Hebrew would enhance your child’s connection to your extended family (e.g. child’s grandparents, cousins, etc.)? (If so, in what way?)
8. What kind of experience do you think your child has when learning Hebrew?
9. In your experience, what would you say are the positive or challenging aspects of your child gaining competence in Hebrew?
10. In your opinion, what does gaining ability in Hebrew mean for your child?
11. If your child would not have learnt Hebrew, do you feel their school/family/community experience would be different? (If yes, how so?)
12. How do you think we can promote positive aspects of bilingualism being harnessed to benefit autistic children?
13. Based on your experiences, what would you recommend to parents of autistic children who are unsure whether their children should learn a second language or not?
14. From your experience, do you find that practitioners make sensible recommendations on whether autistic children should gain a second language?

*Possible prompts: Could you share more about that? How did that feel? Could you give me a few examples?*

## Written Response Open-Ended Questions

Note: Please answer these questions with as much detail and information as possible. If applicable, please provide anecdotes or relate episodes relevant to the questions. If you run out of space, please continue writing on the spare pages at the end of this questionnaire, citing the question number.

1. Can you describe what Hebrew means to **you** and your family?
2. When making the decision on whether to send your child to a Jewish school, how important was the factor of your child gaining ability in Hebrew to you?
3. Can you share what the experience of choosing whether your child should learn Hebrew or not was like?
4. What were the most important factors in making that decision?
5. What was the most important factor influencing your decision on whether your child should learn Hebrew in addition to his/her primary language?
  5a. Why was this factor so important?
6. Did you experience support in your decision-making on this issue from the school, or from professionals such as psychologists or therapists? YES/NO
  6a. If yes, what advice and reasons did the school and/or professionals provide on whether your child should gain ability in a second language such as Hebrew?
  6b. Were you satisfied with the advice given? YES/NO Please explain.
7. Recent research has revealed that children with ASC who learn a second language do not feature more language or developmental delays compared to children with ASC who learn one language. What do you think of this finding?
8. Do you think the school experience of children with ASC is made easier or more difficult if they are taught additional languages (such as Hebrew)? EASIER/MORE DIFFICULT/ OTHER
  Please explain.
9. Do you believe that learning Hebrew would enhance your child’s connection to the wider Jewish community? YES/NO (If yes, in what way?)
10. Do you think that learning Hebrew would enhance your child’s connection to your extended family (e.g. child’s grandparents, cousins, etc.)? YES/NO (If yes, in what way?)
11. What kind of experience do you think your child has when learning Hebrew?
12. In your experience, what are the positive or challenging aspects of your child gaining competence in Hebrew?
13. In your opinion, what would gaining ability in Hebrew mean for your child?
14. If your child would not have learnt Hebrew, do you feel their school/family/community experience would be different? YES/NO (If yes, how so?)
15. How do you think we can promote positive aspects of having ability in two languages (e.g. Hebrew and English) being harnessed to benefit children with ASC?
16. In your opinion, what measures do you think schools should take to best facilitate children with ASC gaining ability in two languages (e.g. in Hebrew and English)?
17. In your opinion what measures do you think practitioners such as therapists or psychologists should take to best facilitate children with ASC gaining ability in two languages (e.g. in Hebrew and English)?
18. Based on your experiences, what would you recommend to **other** parents of children with ASC who are unsure whether their children should learn a second language or not? Please answer as fully as possible.
19. From your experience, do you find that practitioners make sensible recommendations on whether children with ASC should gain a second language? YES/NO
  Please explain:
20. In your opinion do you feel children with ASC should be taught Hebrew in addition to their primary language? YES/NO Please explain your answer.
21. In your experience, do you find that parents and teachers/professionals agree on the issue of whether children with ASC should gain competence in a second language (e.g. Hebrew) in addition to their primary language? YES/NO

Please explain. If no, please also outline where you believe the primary differences in perspectives lie.

## Footnotes

1 Disability-first terms (e.g. autistic person) are used throughout this study, in recognition of the preferences of a majority of autistic people (Kenny et al., 2016) and because person-first language may accentuate stigma towards autistic people (Gernsbacher, 2017). Similarly, autistic people’s preferences regarding terminology surrounding ‘Asperger’s syndrome’ are respected (Sher, 2020).
